# Langerhans Cell Histiocytosis: An Illusion of Hope

**DOI:** 10.5005/jp-journals-10005-1191

**Published:** 2013-04-26

**Authors:** Vela D Desai, Smita R Priyadarshinni, Beena Varma, Rajeev Sharma

**Affiliations:** Head, Department of Oral Medicine and Radiology, Jaipur Dental College, Jaipur, Rajasthan, India; Postgraduate Student, Department of Oral Medicine and Radiology Jaipur Dental College, Jaipur, Rajasthan, India; Associate Professor, Department of Oral Medicine and Radiology Jaipur Dental College, Jaipur, Rajasthan, India; Associate Professor, Department of Oral Medicine and Radiology Jaipur Dental College, Jaipur, Rajasthan, India

**Keywords:** Langerhans histiocytosis, Oral manifestations, Treatment

## Abstract

**Introduction:** Langerhans cell histiocytosis (LCH) is a rare atypical cellular disorder characterized by clonal proliferation of Langerhans cells leading to myriad clinical presentations and variable outcomes. It usually occurs in children and young adults. It can be present with local and systemic manifestation involving skin, bone, mucosal tissues and internal organs.

**Aims and objectives:** The stomatologist plays an important role in management of the disease by keeping in mind the various oral manifestations of the disease.

**Case report:** Of a child with disseminated LCH with multiorgan involvement who presented with failure to thrive, osteolytic bony lesions and extensive cutaneous eruptions.

**Conclusion:** Early diagnosis and awareness is necessary to treat the patients.

**How to cite this article:** Desai VD, Priyadarshinni SR, Varma B, Sharma R. Langerhans Cell Histiocytosis: An Illusion of Hope. Int J Clin Pediatr Dent 2013;6(1):66-70.

## INTRODUCTION

Langerhans cell histiocytosis (LCH) is a proliferative disorder of histiocytes that is characterized by heterogeneous clinical manifestations and an unpredictable course. LCH includes diseases previously designated as histiocytosis X, eosinophilic granuloma, Letterer-Siwe disease, Hand-Schuller-Christian disease, Hashimoto-Pritzker syndrome, self-healing reticulocytosis, pure cutaneous histiocytosis, Langerhans cell granulomatosis, type II histiocytosis and nonlipid reticuloendotheliosis.^1^ It is characterized by a proliferation of abnormal and clonal Langerhans cells in one or more body organs, such as the skin, bone, lymph node, lungs, liver, spleen and bone marrow. The disease can occur at any age, though commonly in infancy or early childhood, often with cutaneous lesions.

Prognostically, it is a confounding disorder with a wide spectrum of outcomes ranging from spontaneous remissions to metastasis and death.

## CASE REPORT

A 4-year-old male patient reported to the outpatient department of Jaipur Dental College with complaint of pain in all teeth since 15 days. Pain was severe, generalized and continuous, associated with swelling in right side of the face. A positive history of recurrent fever after every 3 days since 2 months, accompanied with lethargy and progressive weight loss, was reported by parent.

He was a healthy and well-nourished child at birth without any contributory past medical, dental and family history.

On general physical examination patient was conscious to time, place and person and responded to his mother only. His height and weight was 80 cm and 08 kg respectively [normal range (91-104 cm) and (13-24 kg) respectively] which was less than the normal, this showed the child to be undernourished. He had thin extremities, prominent ribs and pot-shaped belly.

The vital signs were within normal limits. Conjunctiva, oral mucosa and nails showed pallor with yellowish discoloration of sclera, with no signs of clubbing and cyanosis.

He had no pitting pedal edema with only slight enlargement of liver and spleen.

The axillary, inguinal and submandibular lymph nodes were palpable, about 2 to 3 in number, round to oval in shape measuring approximately 1 to 3 × 1 to 2 cms in size. They were firm, tender and slightly mobile. Skin showed multiple papules on the chest, upper abdomen and right thigh measuring 0.1 × 0.1 mm in size. Hairs were thin, spare and golden brown in color and no seborrheic dermatitis was present. Nails showed horizontal brown line near nail bed.

Extraoral examination revealed brachycephalic, frontal bossing with straight profile and incompetent lips.

**Figs 1A and B:**
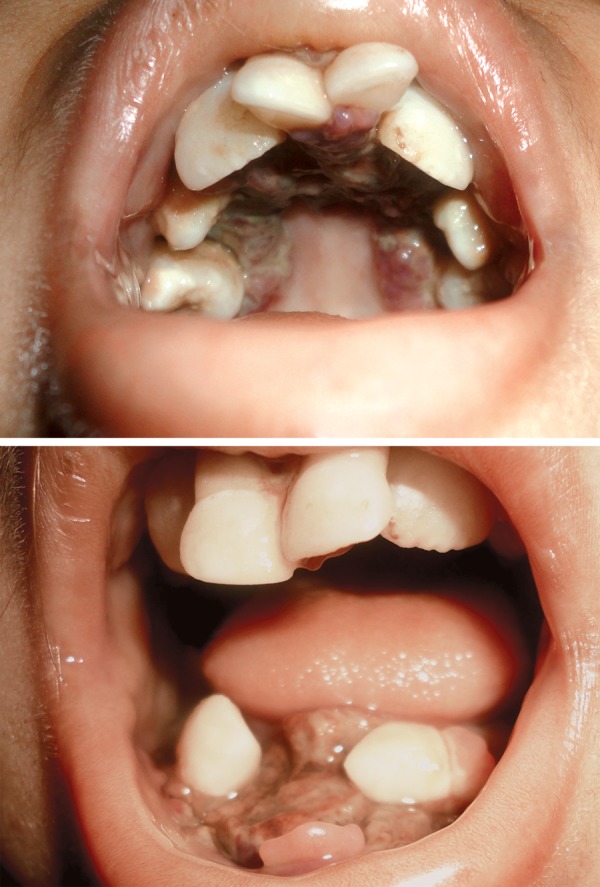
Intraoral findings showing pseudomembranous slough in the maxillary and mandibular arch

Oral hygiene was very poor with fetid odor. The oral mucosa appeared to be pale. The floor of mouth showed greenish yellow pseudomembrane obscuring the alveolar ridge and labial vestibule, loose teeth, along with hyperplasia of gingiva in relation to 41, palate showed similar lesion with hyperplasia of the palatal gingiva which was scrappable and tender on palpation (Figs 1A and B). He had a mixed dentition (11, 51, 53, 21, 63, 73, 75, 83, 85) showing precocious eruption of permanent incisors. Periodontal status of the patient was poor with mobility and recession in maxillary centrals and mandibular canines.

**Fig. 2:**
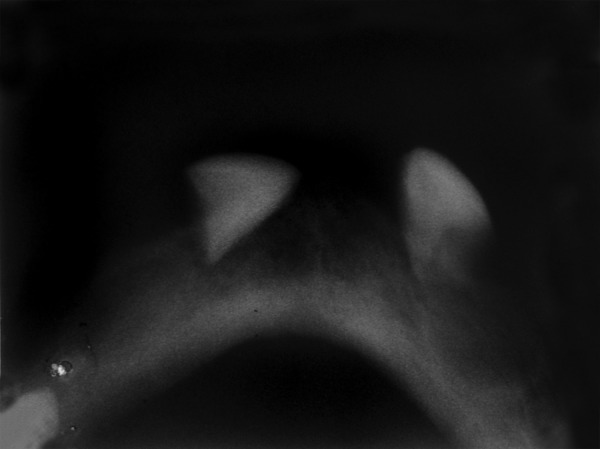
Mandibular occlusal radiographs showing multiple missing teeth along with the permanent tooth buds

With the parents consent, the child was subjected for the hematological and radiographic investigations. The occlusal radiograph showed generalized bone loss giving a floating tooth appearance. There was slight radiolucency with well-defined cortical borders in relation to 85 and 75 with the absence of permanent tooth buds (Fig. 2).

**Fig. 3:**
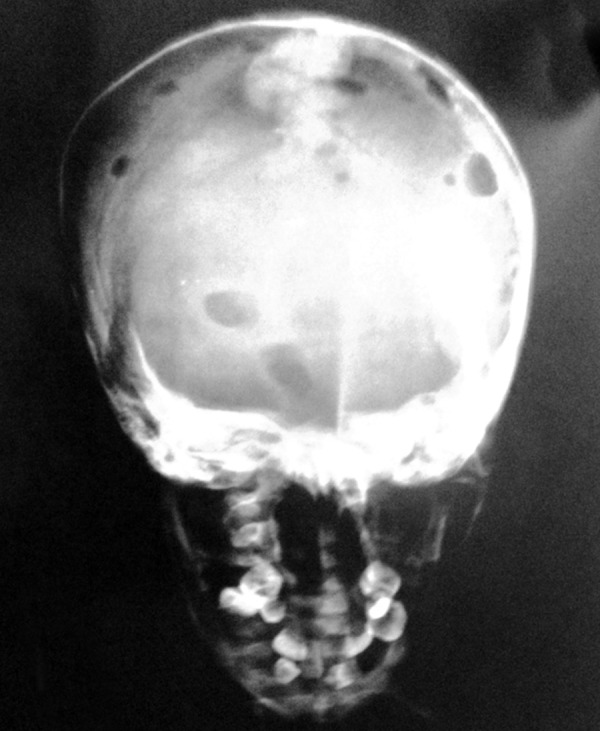
AP skull shows multiple lytic lesions in the frontal and parietal bones

Anterior-posterior (AP) and lateral skull views showed multiple radiolucencies in the skull vault measuring approximately 0.5 to 1 × 0.5 to 1 cm, which had smooth well-defined punched out margins, with hypoplasia of the mandible. Similar lesions were present in the pelvic bone and spine (Fig. 3).

Hematological values were within normal limits except the Hb% (5.2) which was critically reduced. SGOT (54 IU/ ml) and SGPT (42 IU/ml) were slightly elevated.

Blood smear report showed anisopoikilocytosis, hypochromic, micro- and macrocytes along with tear drop cells without presence of any immature cells.

**Fig. 4:**
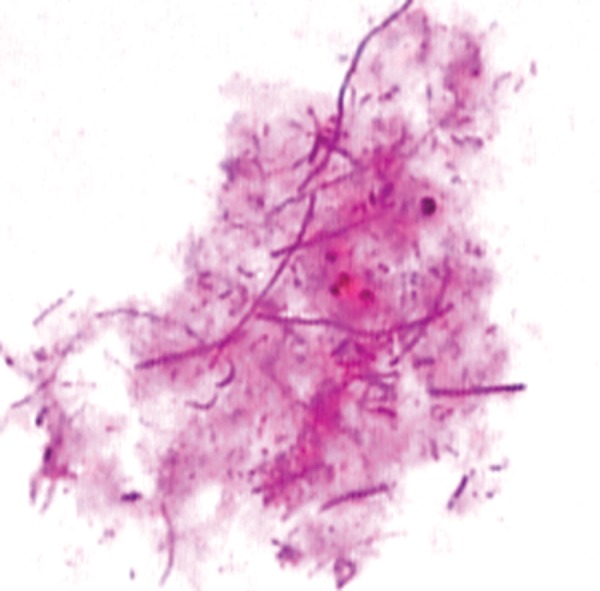
Exfoliative cytology showing multiple candidal hyphae

The histopathological report showed normal cells with numerous eosinophils and histiocytes and numerous chronic inflammatory cells and candidal hyphae (Fig. 4).

Based on the histological and radiographic features a final diagnosis of Langerhans cell histiocyosis (LCH) with multiorgan involvement was given along with anemia and superimposed candidiasis.

**Fig. 5:**
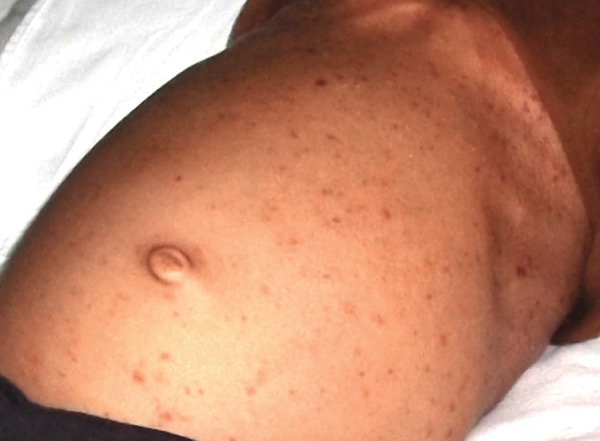
After hospitalization, multiple papules with blotting of stomach

The patient was admitted to the hospital and immediate treatment was started. After 3 days slight improvement was seen in the Hb% (9.3) but platelet count (0.28 lac/cumm) was severely decreased and appearance of multiple purpuric spots seen all over the body, measuring 1 × 1 mm in size (approximately). There was increase in the pedal edema in the right feet and blotting of stomach (Fig. 5).

The bone marrow aspiration report showed normocellular partially diluted marrow showing mild erythroid hyperplasia.

He did not respond to the treatment and succumbed to death after 10 days of his first visit who reported in a terminal stage of LCH with multiorgan involvement.

## DISCUSSION

LCH is a rare disease with diverse forms of clinical presentation ranging from a benign course to diffuse progressive disease.^2,3^ Clinically and histologically the histiocytoses comprise a diverse group of proliferative disorders characterized by the infiltration and accumulation of histiocytes and other effector cells of the immune system within various tissues. The generic term ‘histiocyte’ refers to several types of cells including: Monocytes/macrophages, dermal/interstitial dendritic cells and Langerhans cells (LCs).

The incidence of LCH ranges from 0.5 to 5.4 cases per million persons per year, depending upon the age of the population investigated.^4^ The disease seems to particularly affect young children between 1 and 4 years, with a male predominance.

LCH can involve any bone, but the skull (especially the calvaria and temporal bones), pelvis, spine, mandible, ribs and tubular bones are the commonly involved sites as seen in the present case. In majority of cases with localized osseous lesions LCH is considered a benign process and single bone lesions are known to resolve spontaneously. Whereas multisystem LCH has a poorer prognosis and a fulminate disease process. Several large retrospective studies consisting of neonates and children under the age of 4 have shown that 51 to 71% of children with LCH present with multiorgan disease.^5^

Despite the fact that LCH was first identified at the turn of the 20th century, the etiology remains unknown. The general consensus is that patients with LCH have a deregulated immune response with failed transition from ‘innate’ to ‘adaptive’ immunity.^6^

The LCs in LCH manifests as an activated immune phenotype, resulting in their increased proliferation and migration. Aberrant or uncontrolled cytokine production by these inflammatory cells likely results not only in further proliferation of LCs, but also contributes to the pathological sequelae of LCH, including fever, fibrosis, bone resorption, and necrosis which was also seen in our case.^7^

The cornerstone of diagnosis in LCH includes identification of the characteristic clinical features, but also requires correlating histopathological, radiographic and immunohistochemical findings. But a definitive diagnosis requires the lesional cells to exhibit positive staining showing the number of LCs with identifiable Birbeck granules.

The histiocyte society has established a set of guidelines to assist in the diagnosis and study of LCH.^8^ The initial evaluation consists of a complete physical examination, inclusive of height and weight measurements, in addition to laboratory studies including hematological assays and coagulation studies, liver function tests and urine osmolality. Although some authorities advocate bone marrow examination in every baseline examination, it is not required unless symptoms or blood tests suggest involvement. Lastly, the patient must have a complete skeletal radiographic survey and chest radiography. Patients with identified abnormalities require more specific studies, such as pulmonary function tests and lung biopsy, small bowel series, liver biopsy, panoramic dental films, CT or MRI of the brain with particular attention paid to the hypothalamic-pituitary axis, endocrine evaluation and otolaryngology consultation with audiogram.^8^

At present the treatment of LCH is still controversial due to the rarity of the disease and absence of universally accepted standards. The severe multivisceral forms require much more heavy handed management, usually referred to oncology hematology departments. The therapy aims to switch off the cytokine expression that maintains the proliferation of LCH cells, macrophages and lymphocytes, rather than to eradicate an abnormal cell clone. The risk for mortality predicted at diagnosis can be significantly modified by response to initial therapy. Treatment of LCH may include surgery, radiation therapy, or oral, topical and intravenous medication. The recommended duration of therapy is 6 months for patients who require chemotherapy for bone, skin or lymph node involvement (Tables 1 to 3).

**Table 1:** **Table 1:** Treatment of LCH low-risk disease (single-system or multisystem)

Skin lesions
• Steroids^9^ oral methotrexate (20 mg/m^2^) weekly for 6 months.
• Oral thalidomide 50 to 200 mg nightly.
• Topical application of nitrogen mustard is effective for cutaneous LCH that is resistant to oral therapies, but not for disease involving large areas of skin.
• Psoralen and long-wave ultraviolet radiation (PUVA).
Skull lesions: Frontal, parietal, or occipital regions or single lesions of any other bone:
• Spleen, liver, bone marrow or lung (may or may not include skin, bone, lymph node or pituitary gland).
• Curettage only or curettage plus injection of methylprednisolone, complete excision.
• Skull lesions in the mastoid, temporal or orbital bones, multiple bone lesions; or combinations of skin, lymph node or pituitary gland with or without bone lesions.
• Among 6 to 12 months of vinblastine and prednisone.
• Weekly vinblastine (6 mg/m^2^) for 7 weeks then every 3 weeks for good response.
• Daily prednisone (40 mg/m^2^) for 4 weeks then tapered over 2 weeks .
Afterward, prednisone is given for 5 days at 40 mg/m^2^ every 3 weeks with the vinblastine injections.

**Table 2:** **Table 2:** Treatment of LCH high-risk multisystem disease

Spleen, liver, bone marrow or lung (may or may not include skin, bone, lymph node or pituitary gland).
Cytosine arabinoside, vincristine, and prednisolone followed by 6 months of maintenance therapy with cytarabine, vincristine, prednisolone and low-dose intravenous methotrexate. Patients had a poor response to the initial regimen, they were switched to a salvage regimen of intensive combination doxorubicin, cyclophosphamide, methotrexate, vincristine and prednisolone.


**Table 3:** **Table 3:** Treatment of LCH with CNS involvement

Dexamethasone, 2-CdA, retinoic acid, intravenous immunoglobulin (IV Ig), and cytarabine with or without vincristine have been used.
Retinoic acid was given at a dose of 45 mg/m^2^ daily for 6 weeks, then 2 weeks per month for 1 year.
IV g (400 mg/m^2^) was given monthly and chemotherapy consisting of oral prednisolone with or without oral or intravenous methotrexate and oral 6-mercaptopurine were given for at least 1 year.
Cytarabine 100 mg/m^2^ daily on days 1 to 5 during induction and 150 mg/m^2^ on day 1 of each maintenance cycle (every 2 weeks for 6 months).^8^


To aptly determine a patient's prognosis and treatment protocol, it is currently recommended that patients are risk-stratified based upon the number of organs involved and degree of organ dysfunction.

Although some studies found that the younger the patient is at the time of diagnosis, the worse the prognosis, this only holds true when multiorgan involvement is present, because neonates who present with isolated cutaneous lesions do exceptionally well.

## CONCLUSION

In the paper we report a case of classical histiocytosis X with all the clinical features, investigations and follow-up. Oral physicians play a major role in identifying the patient as they present with the oral findings. These conditions being rare may be the first evidence of much more severe conditions. So early recognition with long-term planning and aggressive preventive activities is necessary for the identification and referral of the patient.
